# Separation, purification, and crystallization of 1,5-pentanediamine hydrochloride from fermentation broth by cation resin

**DOI:** 10.3389/fbioe.2022.1104041

**Published:** 2023-01-04

**Authors:** Hui Li, Xu Chen, Yibo Tang, Yue Yang, Feng He, Xin Wang, Ganlu Li, Kequan Chen, Pingkai Ouyang, Yuning Yang

**Affiliations:** ^1^ College of Biotechnology and Pharmaceutical Engineering, Nanjing Tech University, Nanjing, China; ^2^ Jiangsu Institute of Industrial Biotechnology, JITRI Co., Ltd., Nanjing, China; ^3^ Gansu Yinguang Juyin Chemical Co., Ltd., Baiyin, China

**Keywords:** 1,5-pentanediamine hydrochloride, resin, purification, crystallization, separation

## Abstract

1,5-Pentanediamine hydrochloride (PDAH) was an important raw material for the preparation of bio-based pentamethylene diisocyanate (PDI). PDI has shown excellent properties in the application of adhesives and thermosetting polyurethane. In this study, PDAH was recovered from 1,5-pentanediamine (PDA) fermentation broth using a cation exchange resin and purified by crystallization. D152 was selected as the most suitable resin for purifying PDAH. The effects of solution pH, initial temperature, concentration of PDA, and adsorption time were studied by the static adsorption method. The equilibrium adsorption data were well fitted to Langmiur, Freundlich, and Temkin-Pyzhev adsorption isotherms. The adsorption free energy, enthalpy, and entropy were calculated. The experimental data were well described by the pseudo first-order kinetics model. The dynamic experiment in the fixed bed column showed that under optimal conditions, the adsorption capacity reached 96.45 mg g^−1^, and the recovery proportion of the effective section reached 80.16%. In addition, the crystallization of the PDAH solution obtained by elution proved that the crystal product quality of resin eluting solution was highest. Thus, our research will contribute to the industrial scale-up of the separation of PDAH.

## 1 Introduction

Bio-based polyurethane (PU) coatings have been widely used in recent decades and are gradually replacing petrochemical coatings because of their advantages of low environmental impact, easy access, low cost, and good biodegradability (Noreen et al., 2016; Paraskar et al., 2021; Ma et al., 2022). Bio-based pentamethylene diisocyanate (PDI) has shown excellent properties in the application of adhesives and thermosetting polyurethane, in which approximately 71% of the carbon content was bio-based. In theory, it is possible to replace petrochemical-based hexamethylene diisocyanate (HDI) (Zeng et al., 2022). At present, the industrial preparation of PDI is mainly liquid-phase phosgenation (Li W et al., 2021). The raw material for PDI is 1,5-pentanediamine (PDA), which needs to be obtained from biomass such as feed corn starch through biological fermentation engineering (Wang et al., 2021). In the two-step reaction, PDA initially reacts with a cold phosgene solution to produce 1,5-pentanediamine hydrochloride (PDAH) and carbamate. Phosgene is then further introduced and gradually heated to produce PDI. There are inevitably some problems in this process, such as the production of tar compounds and chloride by-products, and it is difficult to control the particle size and rate in the salt forming process, all of which result in many impurities and reduced yield (Liu et al., 2020; Li et al., 2020). Therefore, the high-quality PDAH can reduce the occurrence of side reactions by the phosgenation reaction, and this can improve the yield and purity of the final PDI (Takahashi et al., 2019).

A variety of technologies have been used to separate and purify products from fermentation broth, including precipitation, solvent extraction, adsorption, distillation, and membrane separation (Chen et al., 2007; Jeon et al., 2014; Szczygiełda and Prochaska., 2020; Hu et al., 2022; Lee and Lee., 2022). However, there are still a large number of bacteria, proteins, residual sugars and inorganic salts in the fermentation broth. Therefore, it is very important to choose an efficient, low pollution and low-cost technology to improve the yield. The advantages of using macroporous resin are strong adsorption capacity, high selectivity, low material cost, high regeneration possibility, and less pollutants (Li C et al., 2021). Commonly used resins are adsorption and ion exchange resins, which utilize a non-specific physical adsorption mechanism and an ion-exchange mechanism, respectively (Xiong et al., 2019). Macroporous adsorption resins are often used for the separation and purification of natural products, such as flavonoids (Dong et al., 2015), phenolic compounds (Park and Lee., 2021), alkaloids (Zhang et al., 2012), anthocyanin (Yang et al., 2022), and antioxidants (Zou et al., 2017). Macroporous ion exchange resins are commonly used to separate amino acids (Dong et al., 2015; Chen et al., 2016; Zhang et al., 2018), lactic acid (Ahmad et al., 2021), nutrients (Ke et al., 2021), succinic acid (Alexandri et al., 2019), and decolorization (Shi et al., 2017) from fermentation broth. However, there are few reports on the separation and purification of bio-based PDAH from pretreated fermentation broth by macroporous ion exchange resin.

Crystallization is an ancient separation process that is usually the last step in the purification process, and its control is crucial. Crystallization is a common and necessary unit operation in the chemical industry, and it is widely used in various industries, from the production of basic materials to complex pharmaceuticals (Ms et al., 2020; Weng et al., 2020). Compared with other purification processes, crystallization has the advantages of high recovery rate, good quality of recovered solid-liquid products, high yield, low energy consumption, good operability, and good stability (Sparenberg et al., 2021). At present, there are many products obtained by separation and purification using macroporous resin from fermentation broth and crystallization, such as bio-based carboxylic acids (Karp et al., 2018), antibiotics (Zheng et al., 2013), and hormones (Xu et al., 2018). In this study, higher purity and more uniform particle size of PDAH were obtained through crystallization, which provided a basis for the industrial production of PDI.

In order to master the separation performance of the resin and understand the basic principle of PDAH separation, this paper studied the influence of ion exchange resin on the static adsorption of PDA, the related adsorption thermodynamic kinetics, the optimization of the separation process, and the penetration and desorption curves of the fixed bed chromatographic column. The cooling crystallization products of the resin desorption solution and other two raw materials were compared. The results indicate that we were able to separate and purity high-quality PDAH through separation and purification, which can be used in the production of PDI.

## 2 Experiment section

### 2.1 Chemicals and reagents

D113, D150, D152, and D155 resins used in experiment are weakly acidic cation exchange resins, which were purchased from Yuan Ye Biotechnology Co., Ltd. (Shanghai, China). The chemical reagents used in the experiment, ethanol, hydrochloric, acetonitrile and trifluoroacetic acid were purchased from Aladdin (Shanghai, China). The PDA fermentation broth and deionized water were provided by our laboratory.

### 2.2 HPLC conditions

A YMC Carotenoid column (250 mm × 4.6 mm, s-5 μm, Grace, Columbia, MD, United States) was used for these experiments with the following conditions: the mobile phase consisted of a mixture of 5% acetonitrile and 0.5% trifluoroacetic acid at a flow rate of 0.8 ml min^−1^. The injection volume was 10 μl. The column temperature was 35°C, and the differential detector (1,290, Agilent Technologies, Santa Clara, CA, United States) temperature was 35°C (Wang et al., 2021).

### 2.3 Static adsorption and desorption experiments

Before use, four cation exchange resins were soaked in three times the volume of ethanol overnight, then washed with three times the volume of 1.0 M hydrochloric acid, 1.0 M sodium hydroxide, and 1.0 M hydrochloric acid. The resin was then washed with deionized water until the washing solution was neutral. The resin was used after filtration.

Static equilibrium adsorption experiments were carried out in a 20°C constant temperature oscillator. Wet resin (10 ± 0.1 g) was added to 30 ml of 100 g L^−1^ PDA fermentation broth in a 100 ml conical flask. The speed of the thermostatic oscillator was set to 200 r min^−1^ and the reaction time was set to 2.0 h; under these conditions, the PDA fermentation liquid reached adsorption equilibrium. After adsorption, the adsorption solution was filtered and the concentration of filtrate was determined by HPLC. The resin was then washed with deionized water three times, and 20 ml 1.0 M hydrochloric acid solution was added for desorption. The flask was held in a 20°C constant temperature oscillator and shaken for 2.0 h. The desorption solution was analyzed by HPLC (Figueira et al., 2022). The resin was screened according to the equilibrium adsorption capacity, desorption capacity, and desorption rate, and these parameters were calculated according to the following formulas:

$$q_{e}=\frac{\left(C_{0}-C_{e}\right)V_{i}}{W}$$ (1)

$$q_{d}=\frac{C_{d}V_{d}}{W}$$ (2)

$$D=\frac{q_{d}}{q_{e}}\times 100\%$$ (3)

where *q*_*e*_ was the equilibrium adsorption capacity, mg g^−1^;*C*_*0*_ was the initial concentration of adsorption solution, mg L^−1^; *C*_*e*_ was the equilibrium concentration of adsorption solution, mg L^−1^; *V*_*i*_ was the total volume of adsorbed solution, L; *W* was the mass of the wet resin, g. *q*_*d*_ was the equilibrium desorption amount, mg g^−1^; *C*_*d*_ was the desorption solution concentration, mg L^−1^; *V*_*d*_ was the volume of desorption solution, L; *D* was desorption rate, %.

### 2.4 Thermodynamic experiments

The static equilibrium adsorption experiment was carried out at 20°C in a constant temperature oscillator. Wet resin (10 ± 0.1 g) was added to 30 ml PDA fermentation liquid of different concentrations (50–200 g L^−1^) in a 100 ml conical flask. The speed of the thermostatic oscillator was set to 200 r min^−1^ and the reaction time was set to 2.0 h; under these conditions, the PDA fermentation liquid reached adsorption equilibrium. The adsorption solution was then filtered, and the concentration of filtrate after adsorption was determined by HPLC at different initial concentrations.

### 2.5 Kinetic experiments

Kinetic adsorption experiments were carried out at different temperatures (20–40°C) with a thermostatic oscillator. Wet resin (10 ± 0.1 g) was added to 30 ml 100 g L^−1^ PDA fermentation broth in a 100 ml conical flask. The speed of the thermostatic oscillator was set at 200 r min^−1^, and 200 μl was taken from the flask periodically for HPLC. The instantaneous concentration was analyzed until adsorption equilibrium was reached, and was calculated according to:

$$q_{t}=\frac{\left(C_{0}-C_{t}\right)V_{i}}{W}$$ (4)

where *q*_*t*_ was the instantaneous adsorption capacity, mg g^−1^; *C*_*0*_ was the initial concentration of adsorption solution, mg L^−1^; *C*_*t*_ was the concentration of adsorption solution at time t, mg L^−1^; *V*_*i*_ was the total volume of adsorbed solution, L; *W* was the mass of the wet resin, g.

### 2.6 Dynamic adsorption and desorption experiment

A certain amount of wet resin was added to the glass adsorption column by the wet method. The inner diameter of the adsorption column was 2.0 cm and the heights were 10.0, 14.0, 20.0, and 30.0 cm. A peristaltic pump was used to control the flow rate of the liquid at the outlet of the column, and a distributed collector was used to collect the sample outflow quantitatively. When the concentration of PDA at the column outlet was equal to the initial concentration of PDA solution, the adsorption experiment was completed. The effluent concentration of different volume sections was determined by HPLC. The penetration curve of PDA on the resin column was drawn with the effluent volume *V* (ml) as the abscissa and the effluent concentration of PDA as the ordinate. The effects of PDA concentration, flow rate, and the H/D on the dynamic penetration curve of PDA were investigated. In dynamic adsorption experiments, the adsorption capacity of the resin unit was determined by integrating the area above the penetration curve (Figueira et al., 2022), and was calculated according to:

$$m_{a}=\int_{0}^{V_{t}}\left(C_{1}-C_{ta}\right)dV$$ (5)

$$Q_{a}=\frac{m_{a}}{m_{s}}$$ (6)

where *C*_*1*_ was the initial concentration of dynamic adsorption, g L^−1^; *C*_*ta*_ was the concentration of PDA effluent at a certain time, g L^−1^; *V*_*t*_ was the outflow volume corresponding to *C*_*ta*_ time, ml; *m*_*a*_ was the mass of total PDA adsorbed by resin column, mg; *m*_*s*_ was the amount of resin used, g; *Q*_*a*_ was the adsorption capacity of PDA per unit of resin, mg g^−1^.

Hydrochloric acid was used as the desorbing agent, a collector was used to collect the desorbing effluent at the outlet of the chromatographic column, measure the pigment OD value of the effluent, and draw the pigment curve with the effluent product *V* (ml) as the abscissa and the effluent pigment OD value as the ordinate and the concentration of PDA in the desorbing effluent was detected by HPLC. The desorption curve of PDA on the resin column was drawn with the effluent volume *V*_*m*_ (ml) as the abscissa and the concentration of PDA effluent as the ordinate. The effects of hydrochloric acid concentration, flow rate, and H/D on the pigment curve and the dynamic desorption curve of PDA were investigated. In order to obtain the eluent with high concentration, we selected the material solution of the section that reached more than 80% of the peak value of the eluent, and calculated these concentrations according to the following equations:

$$m_{b}=\int_{0}^{V_{m}}C_{td}dV$$ (7)

$$m_{c}=0.8C_{\max}\int_{V_{1}}^{V_{2}}dV$$ (8)

$$F=\frac{m_{b}}{m_{c}}\times 100\%$$ (9)

where *V*_*m*_ was the volume of eluent required for complete desorption of PDA, ml; *C*_*td*_ was the concentration of PDA desorption solution at time t, g L^−1^; *m*_*b*_ was the mass of total PDA eluted by resin column, g; *C*_*max*_ was the peak concentration of desorption solution, g L^−1^; *V*_*1*_ was the volume of eluent when the absorption reached 0.8*C*_*max*_ for the first time, ml; *V*_*2*_ was the volume of eluent when the absorption reached 0.8*C*_*max*_ for the second time, ml; *m*_*c*_ was the mass of total PDA 0.8*C*_*max*_ twice by the resin column, g; *F* was the proportion of the mass of total PDA in the section with the concentration above the peak of 80% in the mass of total PDA eluted by resin column,%.

### 2.7 Crystallization of PDAH

The PDAH solution after fermentation (PHF), the PDAH solution decolorization by activated carbon after fermentation (PHDF), and the PDAH solution after elution of resin (PHER) were dissolved into the jacket beaker, preheated to 60°C, then cooled to 20°C–10°C; the crystals were observed 1.0 h after the introduction of crystal precipitates. After the crystallization was complete, the product was extracted by filtration, washed, and dried. The experiment was repeated three times under the same operating conditions to determine the average purity and yield of the product (Sparenberg et al., 2021). A small number of samples were placed on a slide, covered with a cover slide, and observed with an optical microscope and photographed. The purity and yield were calculated according to the following equations:

$$Purity=\frac{m_{1}}{M_{1}}\times 100\%$$ (10)

$$Yield=\frac{m_{1}}{M_{2}}\times 100\%$$ (11)

where *m*_*1*_ was the mass of PDAH actually measured in the final product, g; *M*_*1*_ was the total mass of the final product to be tested, g; *M*_*2*_ was the mass of PDAH in the initial solution, g.

The product structure was analyzed by Nicolet Summit FTIR (Thermo Fisher Technology Co., Ltd. United States). The sample clip was placed in the sample window for infrared scanning determination.

A D8Advance powder X-ray diffractometer (Brock Technology Co., LTD. Germany) was used to detect CuKα rays (1.54056 A) under the following conditions: emission slit 1º; room temperature measuring; working voltage 40 kV; scanning Angle 5–60º; scanning step size 0.05º; scanning speed 1 step/second.

## 3 Results and discussions

### 3.1 Resin selection

**FIGURE 1:**
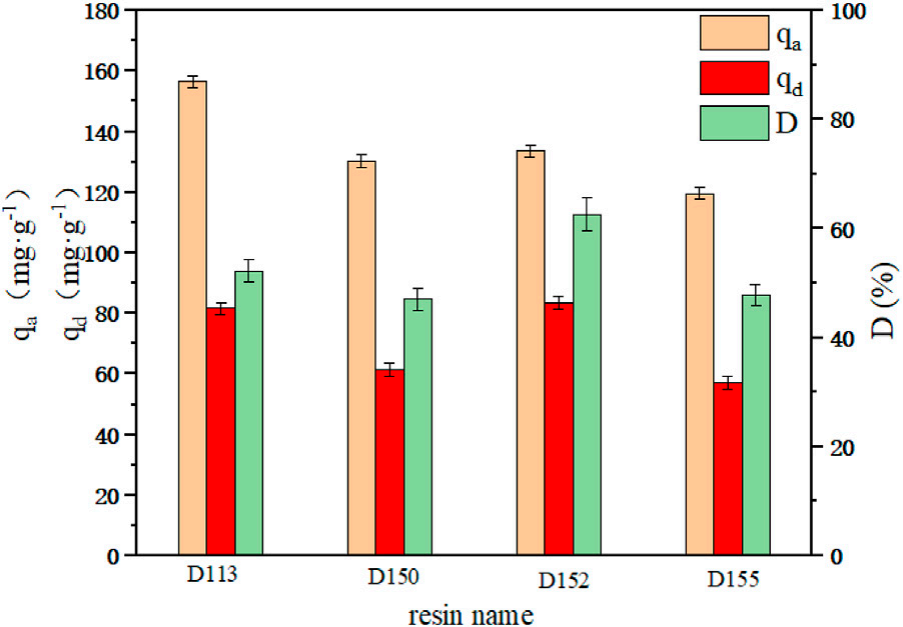
Adsorption and desorption capacity of different cation exchange resins.

The adsorption and desorption capacities of cation exchange resins D113, D150, D152 and D155 are shown in Figure 1. Among the four resins, the unit adsorption capacity of D113 reached the highest of 156.51 mg g^−1^. However, the highest desorption capacity and desorption rate was observed with D152 (83.48 mg g^−1^ and 62.53%, respectively). As can be seen from Table 1, the particle size of D113 was smaller than the other three resins, which increased the contact area between D113 and PDA fermentation broth, and made the adsorption capacity of D113 higher than the other three resins. However, the exchange capacity of D152 was the best among the four resins, which was also the reason why its desorption capacity and desorption rate were higher than the other three resins (Du et al., 2012). Therefore, D152 was selected to further study the adsorption process of PDA fermentation broth.

**TABLE 1:** Different cation exchange resin parameters.

Resins	Matrix	Particle size (mm)	Exchange capacity (mmol g^−1^)	Moisture content (*%*)	Active functional group
D113	Acrylic	0.30–1.2≧95%	10.8	45–52	carboxyl
D150	Acrylic	0.315–1.25≧90%	10	42–50	carboxyl
D152	Acrylic	0.315–1.25≧95%	11.2	50–60	carboxyl
D155	Acrylic	0.45–1.25≧95%	9.5	42–50	carboxyl

### 3.2 Effects of pH and temperature on the adsorption process

**FIGURE 2:**
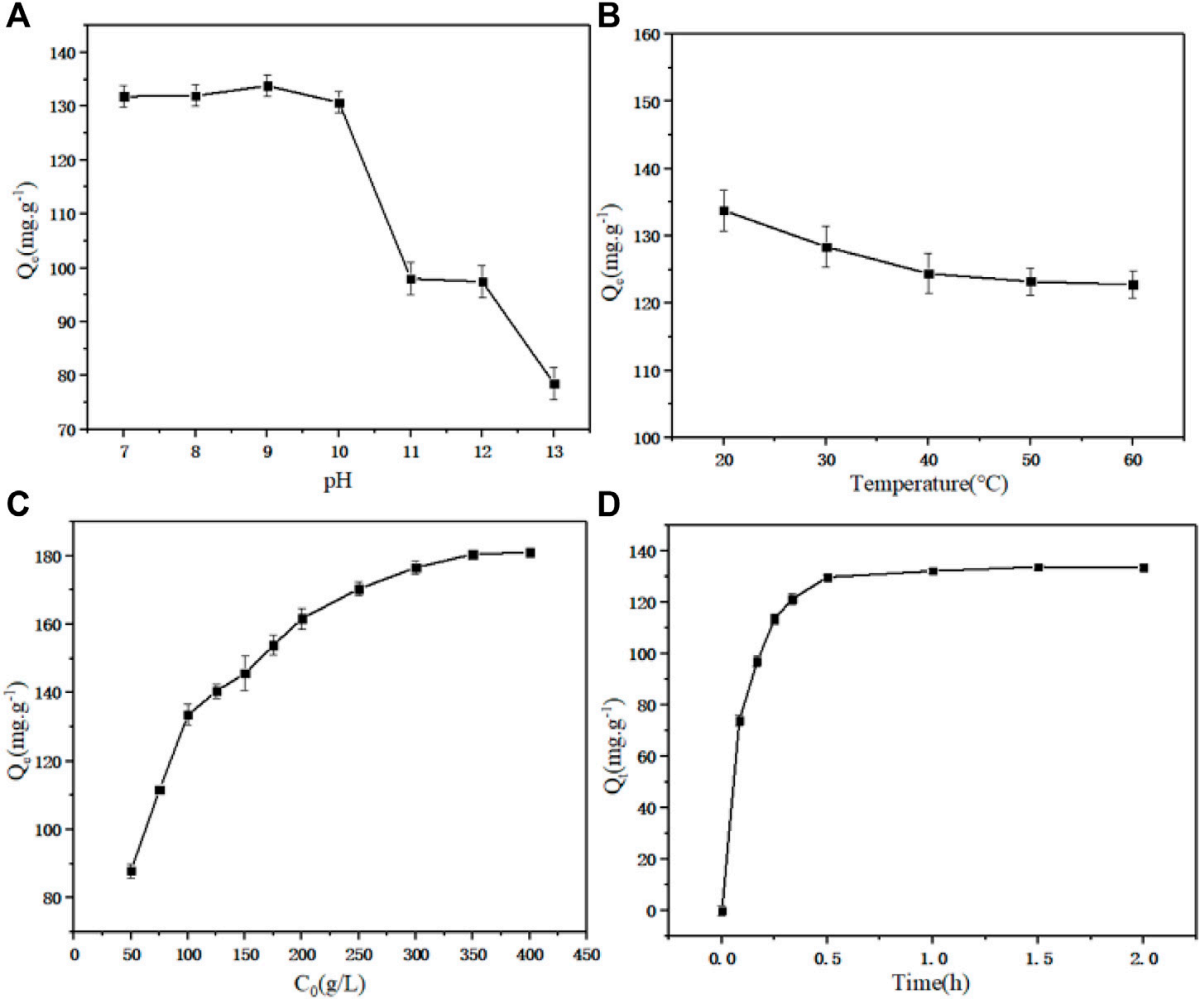
**(A)** Effect of pH on equilibrium adsorption capacity. **(B)** Effect of temperature on equilibrium adsorption capacity. **(C)** Effect of initial concentration of PDA on equilibrium adsorption capacity. **(D)** Effect of adsorption time on equilibrium adsorption capacity.

The equilibrium adsorption capacity of static adsorption under different pH values (7.0–13.0) is shown in Figure 2A. With the increase of pH, the adsorption capacity of D152 for PDA reached 133.80 mg g^−1^ when the pH reached 9.0. There were three main forms of PDA in aqueous solution: C_5_H_14_N_2_, C_5_H_15_N_2_^+^ and C_5_H_16_N_2_^2+^, which can be interconverted by adjusting the pH. When the pH was 9, PDA existed in ionic form, with more C_5_H_15_N_2_^+^ and less C_5_H_16_N_2_^2+^. Under these conditions, it was more conducive to ion adsorption (Lee et al., 2019). When the pH value reached 11, the unit adsorption capacity of PDA decreased significantly. When the pH value was high, the PDA mostly existed in the solution in molecular form. At this time, the adsorption was mostly through non-ionic interactions, which were difficult to exchange through with the ionic resin. The adsorption capacity of D152 to PDA decreased with increasing pH.

The equilibrium adsorption capacity of static adsorption under different temperatures (20–60°C) is shown in Figure 2B. It can be seen that when the temperature increased, the equilibrium adsorption capacity showed a negative correlation trend. The increase of temperature caused the adsorption reaction to move in the opposite direction, since the adsorption process was an exothermic event (Zhuang et al., 2020).

The equilibrium adsorption capacity of static adsorption under different initial PDA concentrations (50–400 g L^−1^) is shown in Figure 2C. At low concentration, the total number of adsorption sites was limited for fixed resin dosage, resulting in decreased adsorption efficiency with increasing of concentration. Before reaching the saturation adsorption capacity, the adsorption capacity increased with increaseing initial concentration (Ren et al., 2020). The saturated adsorption capacity of unit resin was 181.06 mg g^−1^.

The equilibrium adsorption capacity of static adsorption for different adsorption times (0–2.0 h) is shown in Figure 2D. As expected, increasing the contact time between D152 and PDA fermentation broth increased the adsorption capacity to a constant level. In the experimental device, when the adsorption time reached 1 h, the equilibrium adsorption capacity did not increase further; there were a large number of surface adsorption sites on the resin, which were able to accommodate the ion mass transfer in the fermentation broth, thus promoting rapid adsorption. With the saturation of the surface adsorption sites, the influence of time on the adsorption rate decreased until it reached equilibrium (Moghimi et al., 2020).

### 3.3 Adsorption isotherm model

The adsorption performance of D152 for PDA was further tested by the adsorption isotherm model. Finding the best correlation for equilibrium curve was for optimizing the adsorption system. The Langmuir model is based on the following assumptions:① the adsorbate is adsorbed on the surface of the adsorbent as a monolayer; ② Adsorption is dynamic, and the adsorbed molecules will return to the original solution under the influence of thermal motion; ③ There is no interaction between the adsorbate molecules adsorbed on the surface of the adsorbent (Vinco et al., 2022). The following formula was used to calculate the equilibrium absorption capcity:

$$q_{e}=\frac{q_{m}K_{L}c_{e}}{1+K_{L}c_{e}}$$ (12)

where *q*_*e*_ was the equilibrium adsorption capacity, mg g^−1^; *q*_*m*_ was the maximum adsorption capacity, mg g^−1^; *K*_*L*_ was adsorption equilibrium constant, L mg^−1^; *C*_*e*_ was the equilibrium adsorption concentration, mg L^−1^.

The Freundlich model is an empirical adsorption model with non-uniform surface (Ahmad et al., 2021), and can be expressed by the following equation:

$$q_{e}=K_{F}C_{e}^{1/n}$$ (13)

where *q*_*e*_ was the equilibrium adsorption capacity, mg g^−1^; *C*_*e*_ was the equilibrium adsorption concentration, mg L^−1^; *K*_*F*_ was the adsorption capacity, (mg g^−1^)·(mg L^−1^)^1/n^; *n* was the characteristic parameter of the equation.

The Temkin-Pyzhev model assumes a linear, rather than logarithmic, decrease in the heat of adsorption for surface molecules of adsorbents (Foo and Hameed., 2010), and can be described as:

$$q_{e}=\frac{RT}{b_{T}}\ln\left(a^{T}C_{e}\right)$$ (14)

where *q*_*e*_ was the equilibrium adsorption capacity, mg g^−1^; *C*_*e*_ was the equilibrium adsorption concentration, mg L^−1^; *a*_*T*_ was the thermodynamic constant, L mg^−1^; *b*_*T*_ was the thermodynamic constant, J mol^−1^.

**FIGURE 3:**
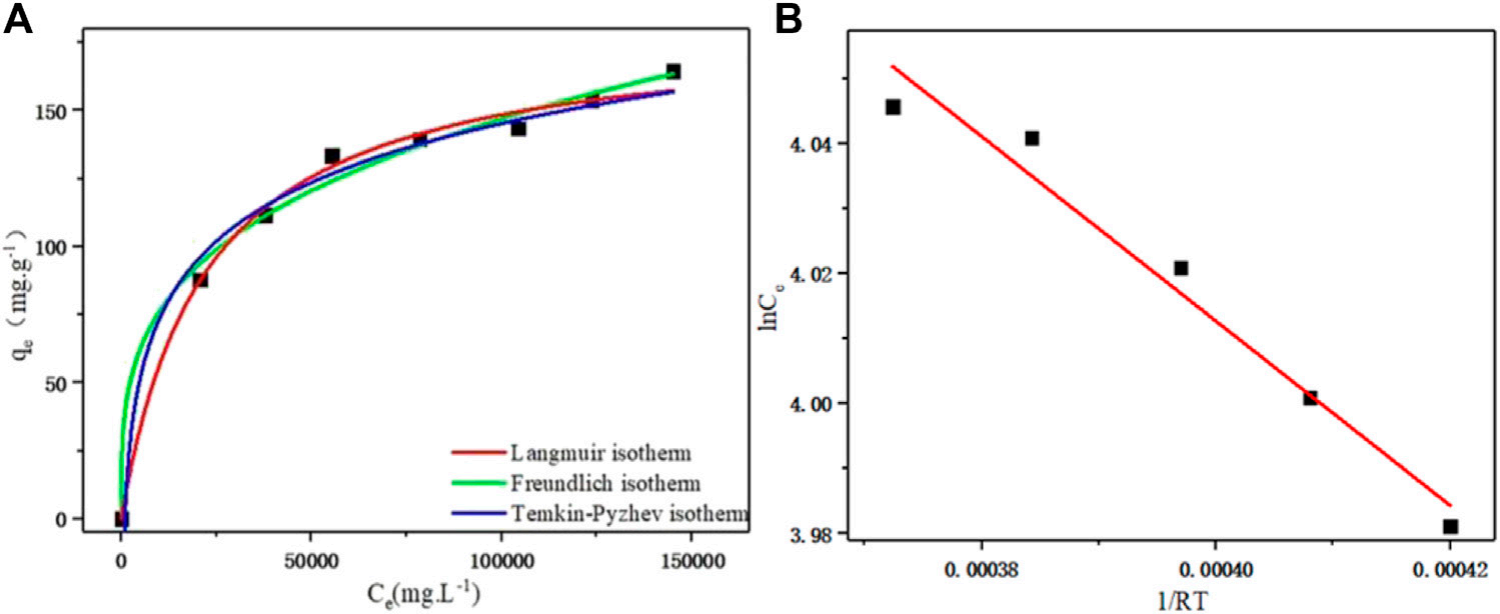
**(A)** Langmiur, Freundlich, and Temkin-Pyzhev adsorption isothermal curves of D152 cation exchange resin for PDA. **(B)** Linear fit of the Clapeyron-Clausius equation.

The fitting of different adsorption isotherm models is shown in Figure 3A. With the increase of PDA equilibrium concentration, the distribution coefficient between the solid phase and liquid phase gradually decreased, and the saturation of resin phase gradually increased. With the increase of the equilibrium concentration, the slope of the equilibrium curve gradually decreased, indicating that the affinity between the exchange ion and the resin decreased with the increase of the solution equilibrium concentration (Hou et al., 2022). The second derivative of the adsorption isotherm (second derivative of f” (C_e_) < 0) showed that the ion exchange equilibrium of PDA on D152 cation exchange resin was favorable.

The basic characteristics of the Langmuir isotherm can be expressed by a dimensionless constant *R*_*L*_ (Zhou et al., 2019):

$$R_{L}=\frac{1}{1+R_{L}C_{0}}$$ (15)

where *R*_*L*_ > 1 indicates that the adsorption was unfavorable, *R*_*L*_ = 1 indicates that the adsorption was linear, and *R*_*L*_ < 1 indicates that the adsorption was favorable. The *R*_*L*_ values under different PDA concentrations were <1, indicating that D152 cation exchange resin had a good adsorption effect on PDA.

In the Freundlich model, when 1/*n* was in the range of 0.1–0.5, the adsorbent was easily adsorbed by the resin, and when 1/*n* was greater than 2.0, the adsorption was inhibited. The results are shown in Table 2. *n* value was 3.335 and 1/*n* value was 0.30, which ranged from 0.1 to 0.5, indicating that the adsorption process of PDA on D152 cation exchange resin was easy to carry out.

**TABLE 2:** Relevant parameters of the Langmiur, Freundlich and Temkin-Pyzhev adsorption isothermal models.

Model	Parameters	D152
Langmiur	*K*_*L*_ (*L* *mg* ^*−1*^)	4.5 × 10^–5^
	*q*_*m*_	181.49
	*R* ^*2*^	0.993
Freundlich	*K*_*F*_ [(*mg* *g* ^*−1*^)*·*(*mg* *L* ^*−1*^)^*1/n*^]	4.671
	*n*	3.335
	*R* ^*2*^	0.989
Temkin-Pyzhev	*a*_*T*_ (*L mg* ^*−1*^)	1.13 × 10^−3^
	*b*_*T*_ (*J mol* ^*−1*^)	78.702
	*R* ^*2*^	0.985

The Temkin-Pyzhev adsorption isotherm model was used to fit the adsorption isotherm data of PDA on D152 resin. As shown in Figure 3A, it can be seen that the fitting effect was good, and the uniform distribution of molecular binding energy of the adsorption layer was deduced (Foo and Hameed, 2010).

It can be seen from Table 2 that the correlation coefficients obtained by fitting the three isothermal models are all good (*R* ^*2*^ > 0.98), but the correlation coefficient (*R* ^*2*^ = 0.993) fitted by the Langmuir isothermal model is better than the Freundlich (*R* ^*2*^ = 0.989) and Temkin-Pyzhev (*R* ^*2*^ = 0.985) models, and the theoretical maximum adsorption capacity of the Langmuir isothermal model was 181.49 mg g^−1^, which was closer to the experimental maximum adsorption capacity of 181.06 mg g^−1^. Therefore, the adsorption data of D152 for PDA was closer to the Langmuir isothermal model. It is concluded that PDA is adsorbed on the surface of D152 resin as a monolayer, and the adsorption process is dynamic. There is no interaction force between PDA molecules adsorbed on the resin surface.

### 3.4 Adsorption thermodynamic parameters

In order to understand the thermodynamic characteristics of PDA adsorption on D152, the thermodynamic parameters of the adsorption process at 20°C were studied. Due to the good correlation of the Freundlich model, the calculation formulas of Gibbs free energy change ( ∆ *G*), enthalpy change ( ∆H ), and entropy change ( ∆S ) parameters were defined as (Chen and Zhang, 2014; Wang et al., 2019):

$$\ln C_{e}=\frac{∆H}{RT}+C_{K}$$ (16)

∆G=−nRT (17)

$$∆S=\frac{∆H-∆G}{T}$$ (18)

where *C*_*K*_ was a constant; *R* was the general gas constant, 8.314 J·(mol⋅K)^−1^; *T* was the thermodynamic temperature, K; *n* is the coefficient of Freundlich equation.

According to the Clapeyron-Clausius Eq. 16, the linear fitting is shown in Figure 3B, and the fitting correlation coefficient was *R* ^*2*^ = 0.965. Finally, the enthalpy change was calculated as ∆H = −1.412 kJ mol^−1^, indicating an exothermic reaction; ∆ *G* = −10.474 kJ mol^−1^, indicating that the adsorption reaction described as spontaneous; *ΔS* = 29.89 J·(mol⋅K)^−1^, indicating that the disorder degree of solid-liquid interface increased (Chen and Zhang, 2014; Guo et al., 2014), and the arrangement of PDA adsorbed on the resin surface was more disordered after adsorption.

### 3.5 Adsorption kinetic model

The pseudo first-order kinetics and pseudo second-order kinetics models, which are widely used in kinetics research, are used to fit kinetic data. The pseudo first-order equation model can be described as:

$$q_{t}=q_{e}\left(1-e^{-K_{1}t}\right)$$ (19)

where *q*_*t*_ was the resin adsorption capacity at *t* time, mg g^−1^; *q*_*e*_ was the equilibrium resin adsorption capacity, mg g^−1^; *t* was the adsorption instantaneous time, min; *K*_*1*_ was the pseudo first-order rate constant, min^−1^.

The pseudo-second order equation model can be described as:

$$q_{t}=\frac{K_{2}q_{e}^{2}t}{1+K_{2}q_{e}t}$$ (20)

where *q*_*t*_ was the resin adsorption capacity at *t* time, mg g^−1^; *q*_*e*_ was the equilibrium resin adsorption capacity, mg g^−1^; *t* was the adsorption instantaneous time, min; *K*_*2*_ was a quasi-second-order rate constant, g·(mg min)^−1^.

**FIGURE 4:**
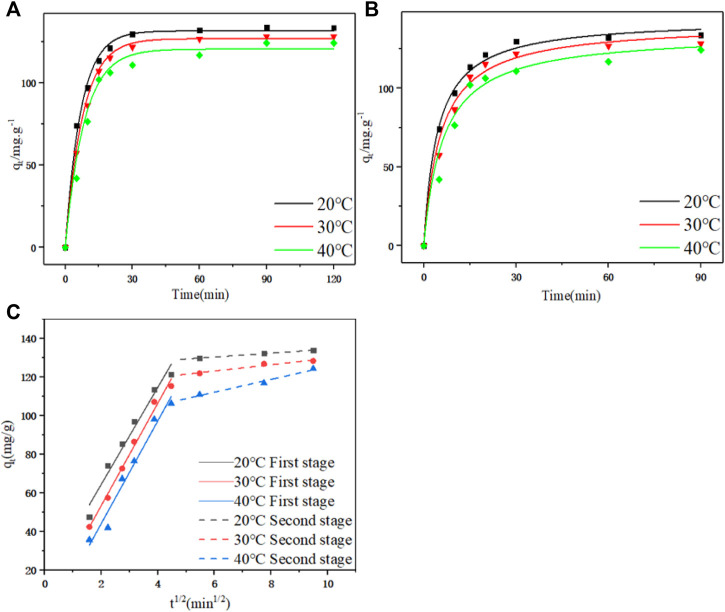
**(A)** Pseudo first-order kinetic curves at different temperatures. **(B)** Pseudo second-order and pseudo first-order dynamic curves at different temperatures. **(C)** Linear fitting diagram of particle diffusion at different temperatures.

According to the fitting parameters in Table 3 and the model fitting diagram in Figures 4A, B, the pseudo first-order kinetics model can better fit the adsorption kinetic data of PDA than the pseudo second-order kinetic model, and the correlation coefficient (*R* ^*2*^) of the pseudo first-order kinetics model was higher than the pseudo second-order kinetics model at different temperatures. The equilibrium adsorption calculated by the pseudo first-order kinetics model was closer to the experimental data, indicating that adsorption may be dominated by ion exchange and other processes and more unit point adsorption. According to the rate constants *K*_*1*_ and *K*_*2*_ obtained by model fitting, the adsorption rate decreased with the increase of temperature, which may be because the adsorption process is an exothermic process (Hou et al., 2022). In this case, the increase of temperature led to the reverse reaction direction of adsorption equilibrium, and reduced the adsorption rate.

**TABLE 3:** Relevant parameters of pseudo first-order and pseudo second-order models at different temperatures.

*T (°C)*	*q*_*e,exp*_ (*mg g* ^*−1*^)	Pseudo-1st-order			Pseudo-2nd-order		
		*q*_*e,cal*_ (*mg g* ^*−1*^)	*K*_*1*_ (*min* ^*−1*^)	*R* ^*2*^	*q*_*e,cal*_ (*mg g* ^*−1*^)	*K*_*2*_ [*g·(mg min)* ^*−1*^]	*R* ^*2*^
20	133.78	131.77	0.1438	0.994	144.27	0.2241	0.994
30	128.37	127.02	0.1277	0.999	141.17	0.1832	0.986
40	124.41	120.70	0.1182	0.987	135.11	0.16	0.968

In order to further analyze the adsorption rate limiting steps on the resin, we also used the particle diffusion model to fit the adsorption kinetic data. The particle diffusion model is as follows:

$$q_{t}=k_{p}t^{1/2}+C$$ (21)

where *q*_*t*_ was the resin adsorption capacity at *t* time, mg g^−1^; *t* was the adsorption instantaneous time, min; *k*_*p*_ was the particle diffusion constant, (mg⋅g^−1^⋅min^1/2^); *C* was the parameter related to boundary layer thickness.

The diffusion mechanism of PDA on D152 was characterized by a particle diffusion model. The curves of *q*_*t*_ and *t* ^*1/2*^ at different temperatures and initial concentrations are shown in Figure 4C. It is clear that these curves show multi-linear graphs, indicating that intra - particle diffusion is not the only rate-limiting step (Zhuang et al., 2020). It can be speculated that the initial stage (0–20 min) was the film diffusion stage, while the later stage (20–90 min) was due to an intra - particle diffusion effect.

The boundary layer thickness related parameter *C* is directly proportional to the range of boundary layer thickness; the greater the value of *C*, the greater the boundary layer effect. If the value of *C* is negative, this indicates that the thickness of the boundary layer delays the diffusion in the particles, while a positive value of *C* indicates that the adsorption is fast (Deng et al., 2020). It can be seen from Table 4 that no matter whether in the film diffusion stage (0 min–20 min) or in the particle diffusion stage (20 min–90 min), the *C* value decreased significantly with the increase of temperature, indicating that the increase of temperature was not conducive to adsorption.

**TABLE 4:** Relevant parameters of particle diffusion model at different temperatures.

*T(°C)*	First stage			Second stage		
	*k*_*p1*_ (*mg g* ^*−1*^ *⋅min* ^*0.5*^)	*C*	*R* ^*2*^	*k*_*p2*_ (*mg g* ^*−1*^ *⋅min* ^*0.5*^)	*C*	*R* ^*2*^
20	25.18	14.02	0.972	1.01	124.37	0.998
30	26.57	0.25	0.990	1.64	113.31	0.974
40	26.65	−9.25	0.970	3.32	92.25	0.990

### 3.6 Dynamic adsorption and desorption results

The dynamic adsorption process of PDA on D152 was investigated under the conditions of single factor variation, different PDA concentration, different flow rate and different height-diameter ratio (H/D), and the penetration curve was used to describe these parameters. The dynamic desorption process of PDA on D152 was investigated under different hydrochloric acid concentrations, flow rates and height-diameter ratios, and desorption curve and pigment curve were used to describe the changes in these parameters and the impact on desorption.

**FIGURE 5:**
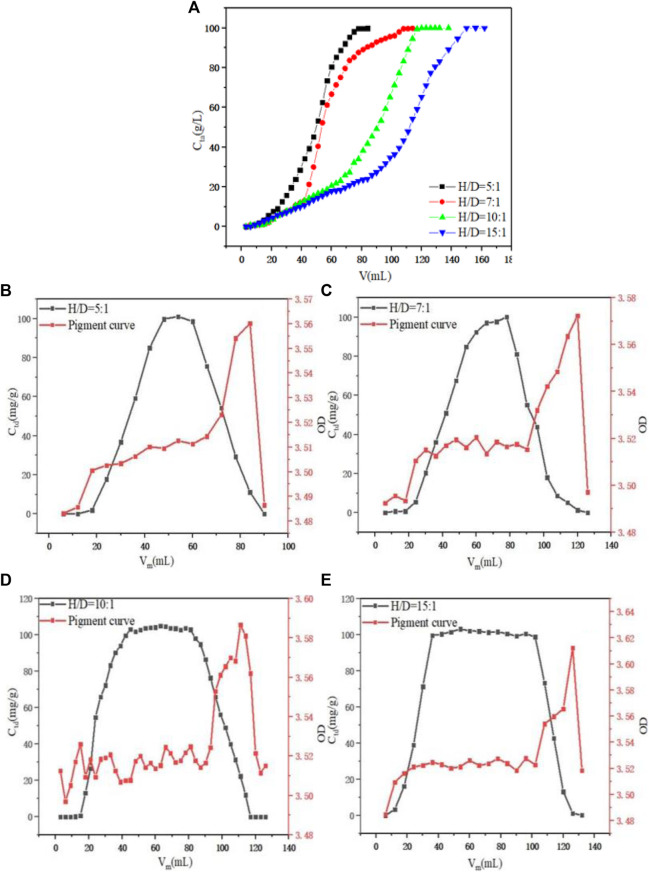
**(A)** Effect of H/D on dynamic adsorption. **(B)** Effect of H/D = 5:1 on dynamic desorption. **(C)** Effect of H/D = 7:1 on dynamic desorption. **(D)** Effect of H/D = 10:1 on dynamic desorption. **(E)** Effect of H/D = 15:1 on dynamic desorption.

Under the condition of a constant flow rate of 1.0 ml min^−1^ and initial concentration of PDA fermentation liquid at 100 g L^−1^, the dynamic adsorption process of PDA on D152 under H/D was investigated, as shown in Figure 5A. When the aspect ratio was 5:1–10:1, the unit adsorption capacity of the resin also increased with the increase of the H/D. The unit adsorption capacity of resin was 87.39, 92.78, 99.68, and 100.26 mg g^−1^, respectively, because the H/D of the column theoretical plate number was larger, and the separation efficiency was improved (Xiong et al., 2019). However, when the H/D reached 10:1, the unit adsorption capacity did not increase significantly. Under the condition of a constant flow rate of 1.0 ml min^−1^ and an initial concentration of desorption solution of 1.5 M HCl, the desorption situation in the elution process was considered. By comparing the F value of the eluted feed solution under different aspect ratios, as shown in Figures 5B–E, the H/D was 5:1–15:1 and it reached 66.85, 75.48, 81.45, and 81.47%, respectively. It can be seen that the F value increased as the H/D increased, but when it reached 10:1, the F value did not change significantly. At the same time, it can be seen that when the section corresponded to the desorption curve was selected, when the H/D was 5:1, 7:1, and 10:1, the OD value was stable at 3.5–3.52, while when the H/D reached 15:1, the OD value exceeded 3.52, and the ability to remove pigment was weaker than the other H/D. Therefore, considering the situation of adsorption and desorption comprehensively, when the H/D was too large, industrial scale up will lead to high operating pressure and higher equipment cost. Therefore, the H/D of 10:1 was selected as the best ratio.

**FIGURE 6:**
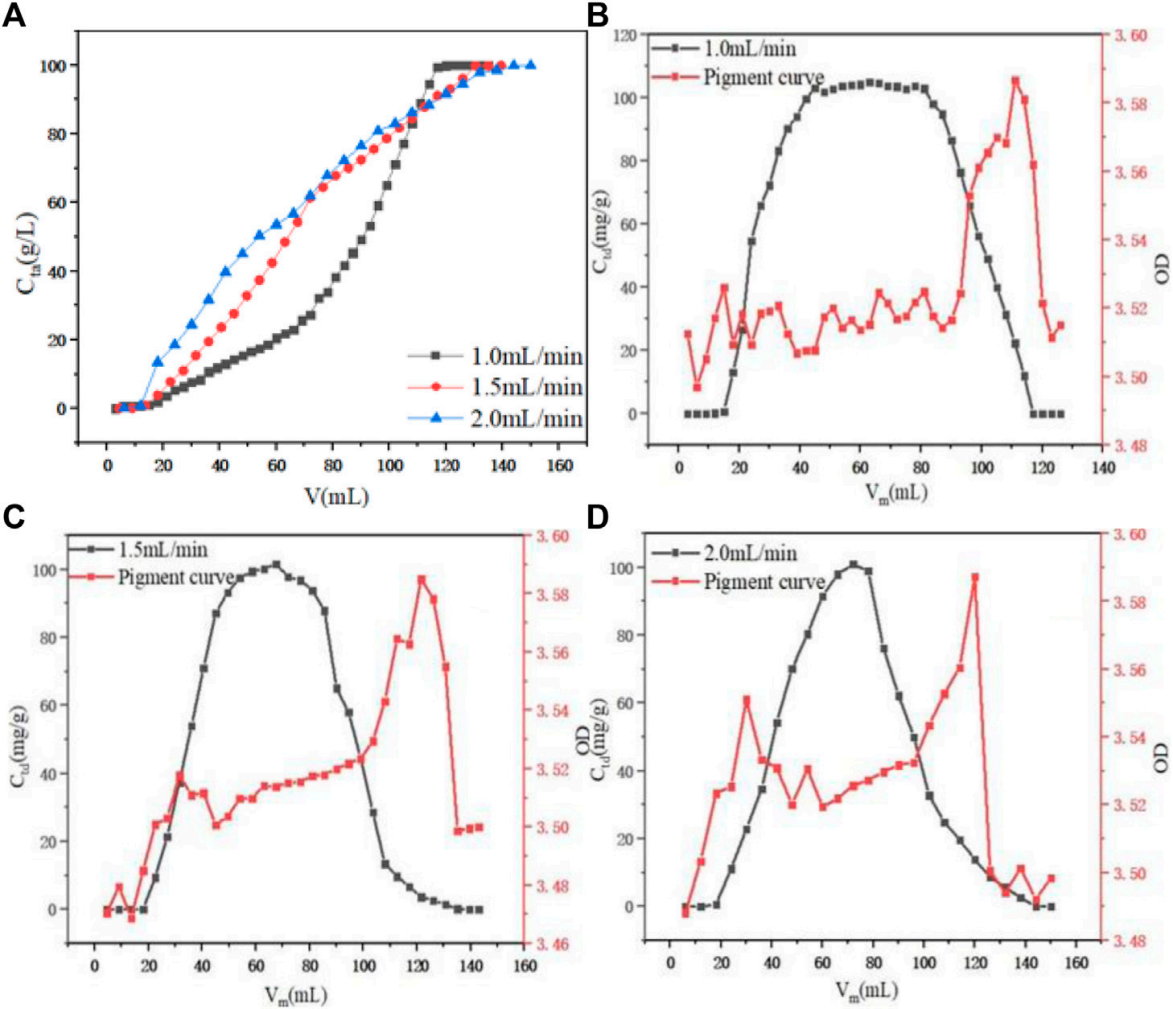
**(A)** Effect of flow rate on dynamic adsorption. **(B)** Effect of 1.0 ml min^−1^ on dynamic desorption. **(C)** Effect of 1.5 ml min^−1^ on dynamic desorption. **(D)** Effect of 2.0 ml min^−1^ on dynamic desorption.

When the aspect ratio was fixed at 10:1 and the initial concentration of PDA fermentation liquid was 100 g L^−1^, the dynamic adsorption process of PDA on D152 resin under different flow rates was investigated. As shown in Figure 6A, with the increase of flow velocity, component adsorption was quickly initiated, but when adsorption was complete, the required volume was significantly increased. The adsorption capacity of 1.0 ml min^−1^ at low flow rate was 96.48 mg g^−1^, while at flow rates of 1.5 ml min^−1^ and 2.0 ml min^−1^, the adsorption capacity was diminished to 88.71 and 77.64 mg g^−1^, respectively. It was inferred that when the flow rate increased, the contact residence time between fermentation liquid and resin decreased, resulting in inadequate adsorption and decreased adsorption capacity (Zhuang et al., 2020). When considering desorption in the elution process when the H/D was fixed at 10:1 and the initial concentration of desorption solution HCl was 1.0 M, as shown in Figures 6B–D, the desorption flow rate increased and the amount of desorption agent required for complete desorption increased, and the elution peak had a certain trailing phenomenon. When the flow rate was 1.0 and 1.5 ml min^−1^, the F value did not differ much, reaching 81.49 and 82.01%, respectively. However, when the flow rate rose to 2.0 ml min^−1^, the F value decreased significantly to 66.43%. This observation may be due to the excessive flow rate, resulting in too fast discharge in the desorption process. After C_max_ was reached, the concentration of feed liquid decreased rapidly. With the increase of the flow rate from 1.0 to 2.0 ml min^−1^, the overall OD value of the corresponding section of the pigment curve was also increased, indicated that 1.0 ml min^−1^ was the best flow rate to removed pigment. Therefore, 1.0 ml min^−1^ was selected as the best flow rate in combination with adsorption.

**FIGURE 7:**
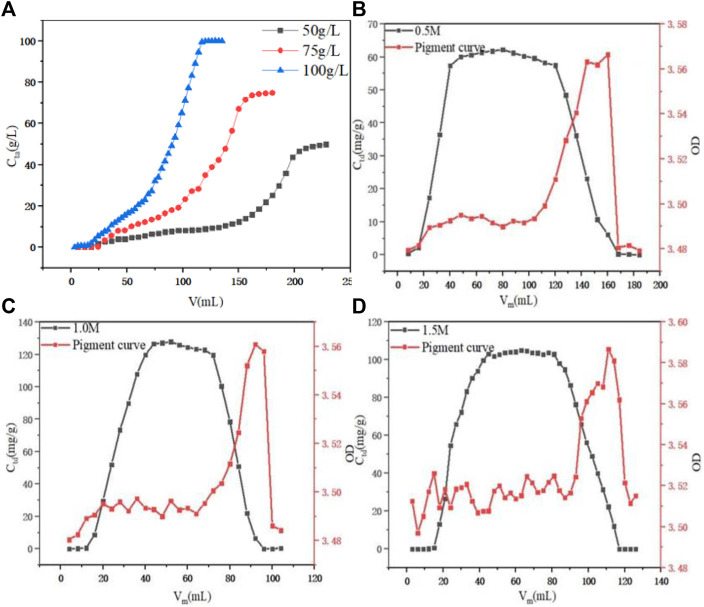
**(A)** Effect of initial PDA concentration on dynamic adsorption. **(B)** Effect of initial 0.5 M HCl on dynamic desorption. **(C)** Effect of initial 1.0 M HCl on dynamic desorption. **(D)** Effect of initial 1.5 M HCl on dynamic desorption.

When the initial PDA fermentation liquid concentration was optimized with a fixed H/D of 10:1 and a constant flow rate of 1.0 ml min^−1^, as shown in Figure 7A, it was found that as the initial PDA fermentation liquid concentration increased from 50 g L^−1^ to 100 g L^−1^, the penetration volume decreased. This may be due to the slow mass transfer process and that the breakthrough time of the low concentration was delayed. This observation was reinforced by the accompanied leftward deviation of the penetration curve, and an increase in the slope of the curve as PDA concentration increased, leading to the reduction of the mass transfer interface (Show et al., 2022). The final adsorption capacity was 76.54, 94.85, and 96.45 g L^−1^ with the initial PDA fermentation liquid concentration was 50, 75, and 100 g L^−1^, respectively. There was little difference between the initial concentration of 75 g L^−1^ and the equilibrium adsorption capacity of 100 g L^−1^, but the required amount of 100 g L^−1^ was lower and the time required for complete penetration was shorter. Therefore, 100 g L^−1^ was selected as the best fermentation liquid concentration.

The concentration of hydrochloric acid was optimized, as shown in Figures 7B–D; it was found that when the concentration of hydrochloric acid was 0.5, 1.0, and 1.5 M, the F value of the eluted liquid was 80.78, 80.16, and 81.49%, respectively, which were relatively close. However, when the concentration of hydrochloric acid was 1.0 M, the maximum concentration value of the eluent reached 127.82 g L^−1^, the overall peak pattern was good. With the concentration of hydrochloric acid increasing from 0.5 M to 1.0 M, the overall OD value of the corresponded section of the pigment curve was stable at 3.48–3.50, while when the concentration of hydrochloric acid reached 1.5 M, the overall OD value of the section was 3.50–3.52, increased significantly. Therefore, the 1.0 M hydrochloric acid was selected as the best elution concentration.

### 3.7 PDAH crystallization

**FIGURE 8:**
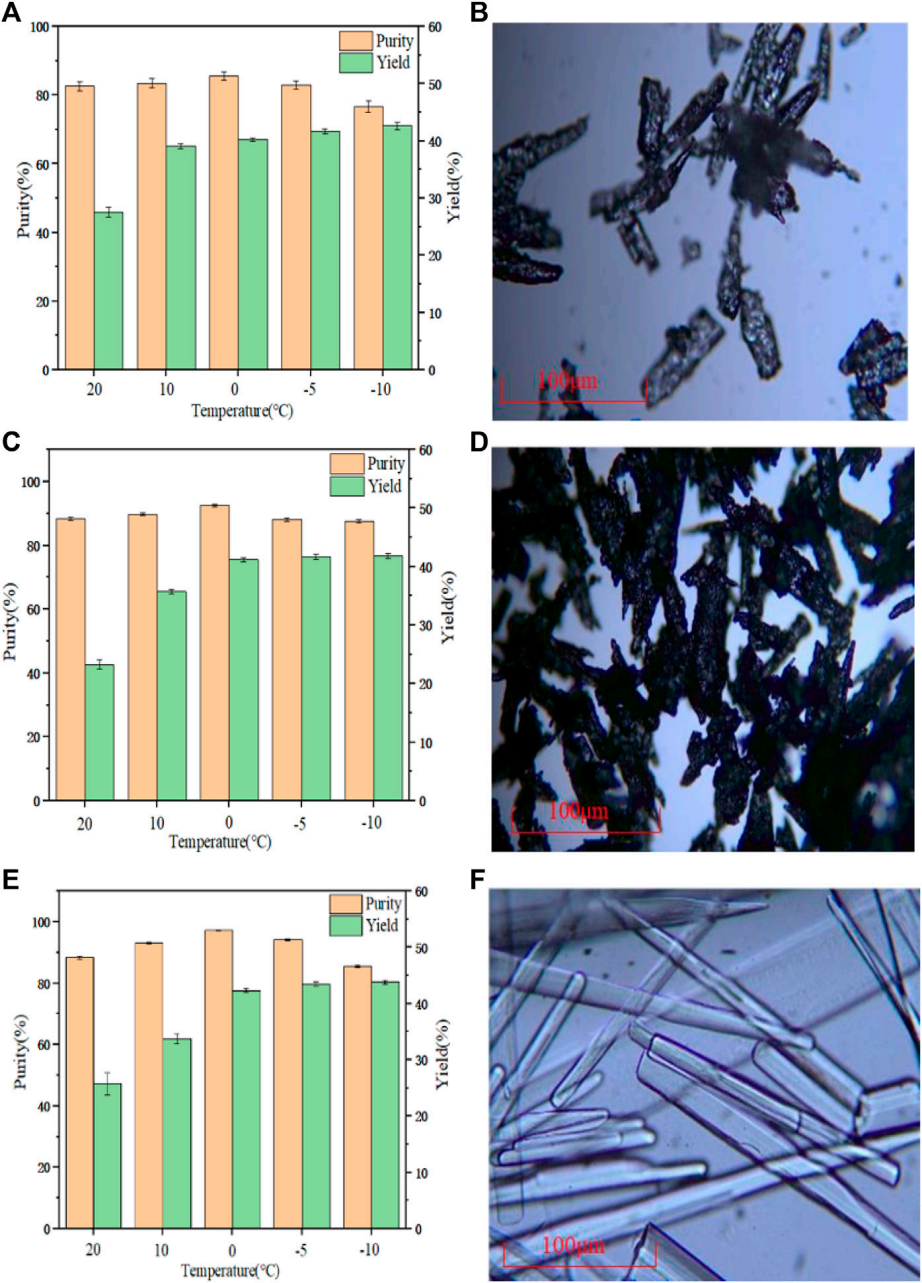
**(A)** Crystallization yield and purity of PHF **(B)** Microscope image of PHF **(C)** Crystallization yield and purity of PHDF **(D)** Microscope image of PHDF **(E)** Crystallization yield, purity of PHER **(F)** Microscope image of PHER.

The cooling crystallization experiment was carried out under the same conditions of three different raw materials, and the results are shown in Figures 8A, C, E. When the temperature was reduced to 0°C, the purity of the PHF, PHDF and PHER was the highest with 85.55, 92.75, and 97.23% respectively. This result may be because when the temperature was lower than 0°C, although the yield was improved, more impurities precipitated, and it was more difficult to separate in the suction filtration process. It can be seen from Figure 8B that there were many impurities in the PHF. Figure 8D shows that the crystal form of PHDF was more complex, while it can be seen from Figure 8F that the crystal form of PHER was long rod type. Therefore, PHER was used for crystallization. The water content of PDAH obtained by Karl Fischer was 0.8%, and the molar ratio of PDA to HCl was 1:1.81 in the obtained PADH crystals which was determined by element analysis.

**FIGURE 9:**
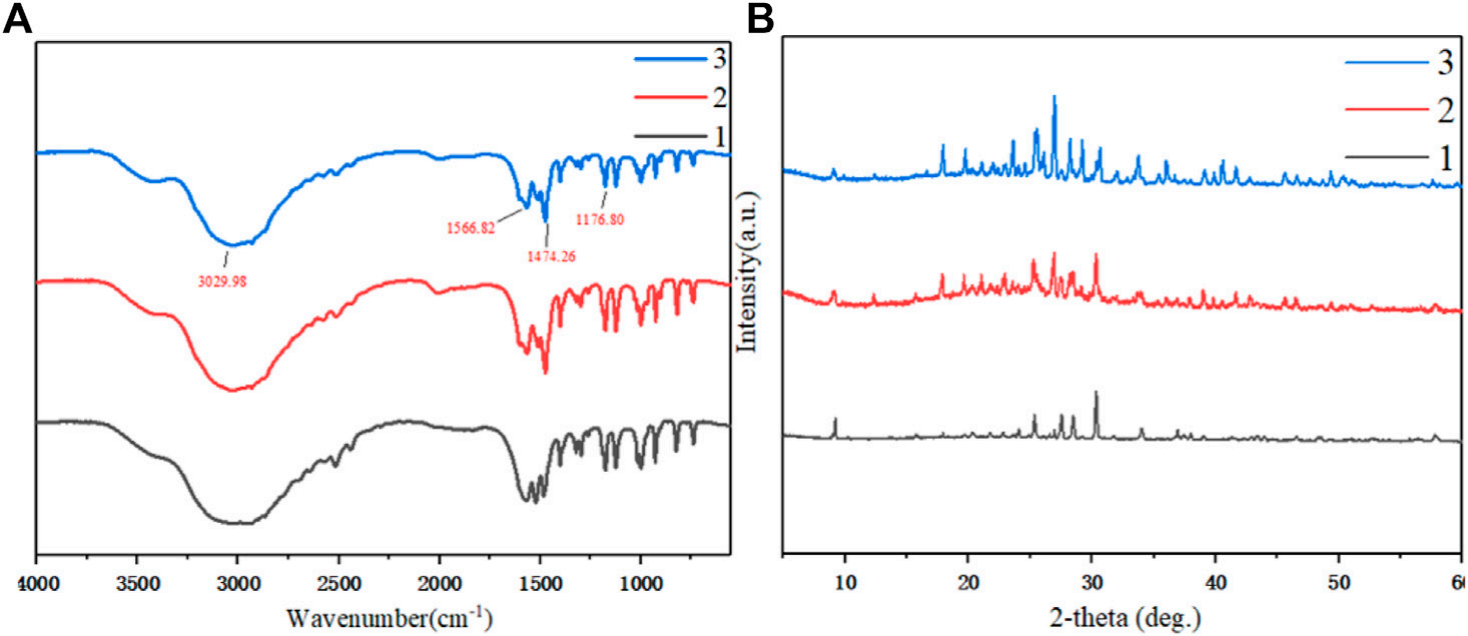
**(A)** Infrared spectrum characterization of cooling crystallization products from three different raw materials **(B)** PXRD characterization of cooled crystalline products from three different raw materials 1. Crystallization of PHF 2. Crystallization of PHDF 3. Crystallization of PHER.

The cooling crystallization products of three different raw materials were characterized by infrared spectroscopy. As shown in Figure 9A, when the amine was salted, the stretching vibration absorption peak of the N-H group shifted significantly to a lower frequency, overlapped with the stretching vibration absorption peak of the C-H bond, and formed a wide and strong spectral band in the range of 3,200–2,200 cm^−1^. Due to the deformation and vibration of the N-H group, the band has a strong absorption peak at 1,600–1,510 cm^−1^. The C-H group had an absorption peak near 1,475 cm^−1^ due to a deformation vibration. The C-N group had a stretching vibration peak at 1,230–1,050 cm^−1^. Through infrared spectrum analysis, it can be confirmed that the main groups of the material structure are basically the same as PDAH. In addition, in the three raw materials, the impurity peak of PHER was less, indicating that its purity was higher.

The products obtained by cooling and crystallization of different raw materials were characterized by PXRD, as shown in Figure 9B. The main peaks of X-ray powder diffraction 2θ = 9.06º, 17.96º, and 25.52º appeared in the same position. These peaks belong to the same crystal form, and the main peak of PHER was sharper, the impurity peak was diminished, and the relative crystallinity was higher (Liu et al., 2020).

## 4 Conclusion

In this study, the adsorption and desorption properties of D152 for PDA in fermentation broth were the best. The Langmiur, Freundlich, and Temkin-Pyzhev equations all fit well with the adsorption equilibrium data of PDA on D152 at 20°C. The adsorption free energy, enthalpy, and entropy were calculated. The results showed that the adsorption of PDA on D152 was a spontaneous exothermic process. The pseudo-first-order model best described the adsorption kinetics of PDA on D152. The dynamic experiment in a fixed bed column showed that the desorption capacity reached 96.45 mg g^−1^, and the F value reached 80.16%. The cooling crystallization of three kinds of raw materials showed that the resin eluting crystallization product had higher quality, the purity reached 97.23%, and the yield was 42.32%. This study provides a low-cost and efficient method for the separation and purification of PDAH from PDA fermentation broth, and contributes to the industrial scale-up of the separation of PDAH.

## Data Availability

The original contributions presented in the study are included in the article/Supplementary Material; further inquiries can be directed to the corresponding author.
